# Unlocking the future of food with the new paradigm of plant-based proteins

**DOI:** 10.3389/fnut.2026.1818969

**Published:** 2026-05-25

**Authors:** Miriana De Feo, Amalia Conte, Matteo Alessandro Del Nobile

**Affiliations:** 1 Department of Humanistic Studies, Letters, Cultural Heritage, Educational Sciences, University of Foggia, Foggia, Italy; 2 Department of Economics, Management and Territory, University of Foggia, Foggia, Italy

**Keywords:** functional properties, plant-based analogs, precision protein engineering, protein bioavailability, sustainability

## Abstract

The global food system faces an unprecedented transformation, fuelled by the dual pressures of increasing consumer demand for high-protein diets, vital for metabolic health and muscle maintenance, and the critical environmental necessity to shift away from resource-intensive animal agriculture. This requires a systemic pivot toward sustainable plant-based proteins. While legumes and cereals offer compelling ecological and nutritional benefits, their widespread integration is significantly hindered by technological and sensory limitations; native plant protein structures often negatively impact the rheology of fortified cereals (weakening the gluten network) and lack the specific functional properties (e.g., coagulation, fibrillization, emulsification) required for high-fidelity meat, dairy, and seafood analogs. This review asserts that the next decade of food innovation will be defined by precision protein engineering. Future research must move beyond simple substitution to focus on the targeted modification of plant proteins using advanced enzymatic, physical, and biotechnological methods. This approach is essential to create high-performance, functional ingredients capable of achieving sensory parity with conventional products, eliminating off-flavors, securing superior textures (lamellarity in fish, meltability in cheese), and ensuring structural stability across diverse food matrices (acidic beverages, high-shear doughs). Crucially, this technological evolution must be coupled with rigorous verification of bioavailability and ecological transparency to fully deliver on the promise of a functional, accepted, and truly sustainable protein supply.

## Graphical Abstract

**Figure:**
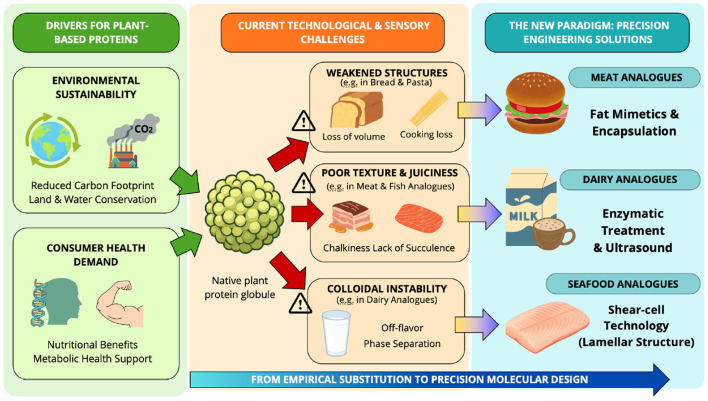

## Introduction

The food industry is currently navigating a period of profound and irreversible transformation, centrally characterized by the rapid and pervasive rise of protein-fortified foods as a dominant global market trend. This fundamental shift is driven by escalating consumer health consciousness, which increasingly recognizes the multifaceted and indispensable benefits of higher protein intake across the lifespan. The scientific evidence is robust, pointing clearly to the value of these products in metabolic health management, particularly their remarkable efficacy in enhancing satiety and providing superior caloric control, a cornerstone of effective weight management strategies (1, 2). Further depth is added by studies showing how the specific role of proteins and amino acids directly influences the central nervous system's control of appetite, underscoring protein's efficacy far beyond simple caloric replacement (3). Beyond the immediate concerns of weight and appetite regulation, this nutritional approach is also indispensable for mitigating age-related sarcopenia, actively supporting the critical maintenance of skeletal muscle mass and functional independence (4). This surging global demand for protein, however, is being critically constrained by a pressing imperative for sustainability. The environmental and ethical costs associated with conventional animal agriculture are now globally recognized, necessitating an urgent, systemic pivot in food production (5). The sheer scale of industrial livestock farming exerts immense pressure on planetary boundaries, particularly in terms of greenhouse gas emissions, agricultural land use, and consumption of water resources. The academic literature is categorical: maintaining the global food system within safe environmental limits requires a drastic reduction in the consumption of meat and dairy products, massively favoring plant-based alternatives (6). Analysis of product life cycles has shown that the choice of protein source is the most powerful factor available to consumers for reducing their ecological impact. The shift from animal to plant products is an irreplaceable environmental mitigation factor, making this transition not just a dietary option, but a critical ecological necessity for humanity (7).

Consequently, the food market is rapidly pivoting toward plant-based proteins, which are lauded not merely as substitutes, but as a critical, more environmentally benign alternative for the future of nutrition and planetary health. The transition toward plant-based protein products is closely aligned with multiple United Nations Sustainable Development Goals (SDGs), as it supports environmental sustainability, public health, and global food security. Encouraging their production and consumption can be a key strategy for achieving a more sustainable future. In particular, the SDGs are: SDG 2 (Zero Hunger) because pant-based proteins are generally more resource-efficient to produce than animal-based proteins. They can help increase food availability and affordability, contributing to global food security and reducing hunger; SDG 3 (Good Health and Wellbeing) because diets rich in plant-based proteins are associated with lower risks of chronic diseases such as heart disease, obesity, and diabetes, supporting healthier populations; SDG 12 (Responsible Consumption and Production) because producing plant-based protein products typically requires fewer natural resources (land, water, energy) and generates lower environmental impact, promoting sustainable consumption patterns; SDG 13 (Climate Action) because plant-based proteins have a significantly lower carbon footprint compared to animal agriculture. Shifting toward these products helps reduce greenhouse gas emissions and mitigates climate change; SDG 15 (Life on Land) because reducing reliance on livestock farming decreases deforestation, land degradation, and biodiversity loss, helping protect terrestrial ecosystems.

In summary, the journey of plant proteins from commodity crop to high-value food ingredient is defined by a dynamic interplay between nutritional science, consumer demand, and technological innovation. This review, therefore, provides a critical and comprehensive analysis of the technological landscape shaping the next generation of protein-fortified foods. We delineate the fundamental challenges inherent in incorporating plant proteins across major food categories, spanning cereals, meat and dairy analogs, and specialized products, and critically evaluate the cutting-edge strategies developed to enhance their intrinsic functional properties. Ultimately, we seek to map the trajectory from raw plant ingredient to high-performance food component, highlighting the essential research gaps and the future opportunities that will define a truly sustainable, functional, and healthy food landscape for the decades to come.

## Methods

This work constitutes a critical and comprehensive review of the current literature, focusing on the intersection between plant protein science and advanced food engineering. The bibliographic search was systematically conducted using major electronic databases, including Scopus, Web of Science, and Google Scholar. Search terms, which were frequently combined, included: “Precision Protein Engineering,” “Plant-Based Analogs,” “Functional Properties,” “Protein Modification,” “High Moisture Extrusion (HME),” “Enzymatic Modification,” “DIAAS,” and “LCA” (Life Cycle Assessment). The selection process prioritized recent, peer-reviewed scientific literature (predominantly published between 2014 and 2025), reflecting the rapid advancement in protein modification technologies. Articles (Original Research, Reviews, and patents) were chosen based on their provision of experimental data or critical analysis regarding the structural modification of plant proteins and the subsequent impact on functional properties, nutritional quality, and sustainability in food product applications. Inclusion and exclusion criteria are used to select only the most relevant and reliable studies about this specific subject with a focus on evidence specifically related to plant-based diets and proteins. Among the inclusion criteria in the plant-based proteins context we selected studies focusing on plant-based protein sources; studies analyzing outcomes like nutritional quality, health effects, and sustainability; specific study designs; publications in a defined time range (last 10 years) and articles written in English. Among the exclusion criteria we considered not suitable studies dealing with applications of plant-based proteins to other fields than food; articles with insufficient data or unclear methodology; non-scientific publications (e.g., opinion pieces, blogs) and duplicate studies. The synthesis of this information has been organized to provide a critical assessment and delineate future research directions within the field.

## Strength points and limitations of plant-based proteins

**Figure 1:**
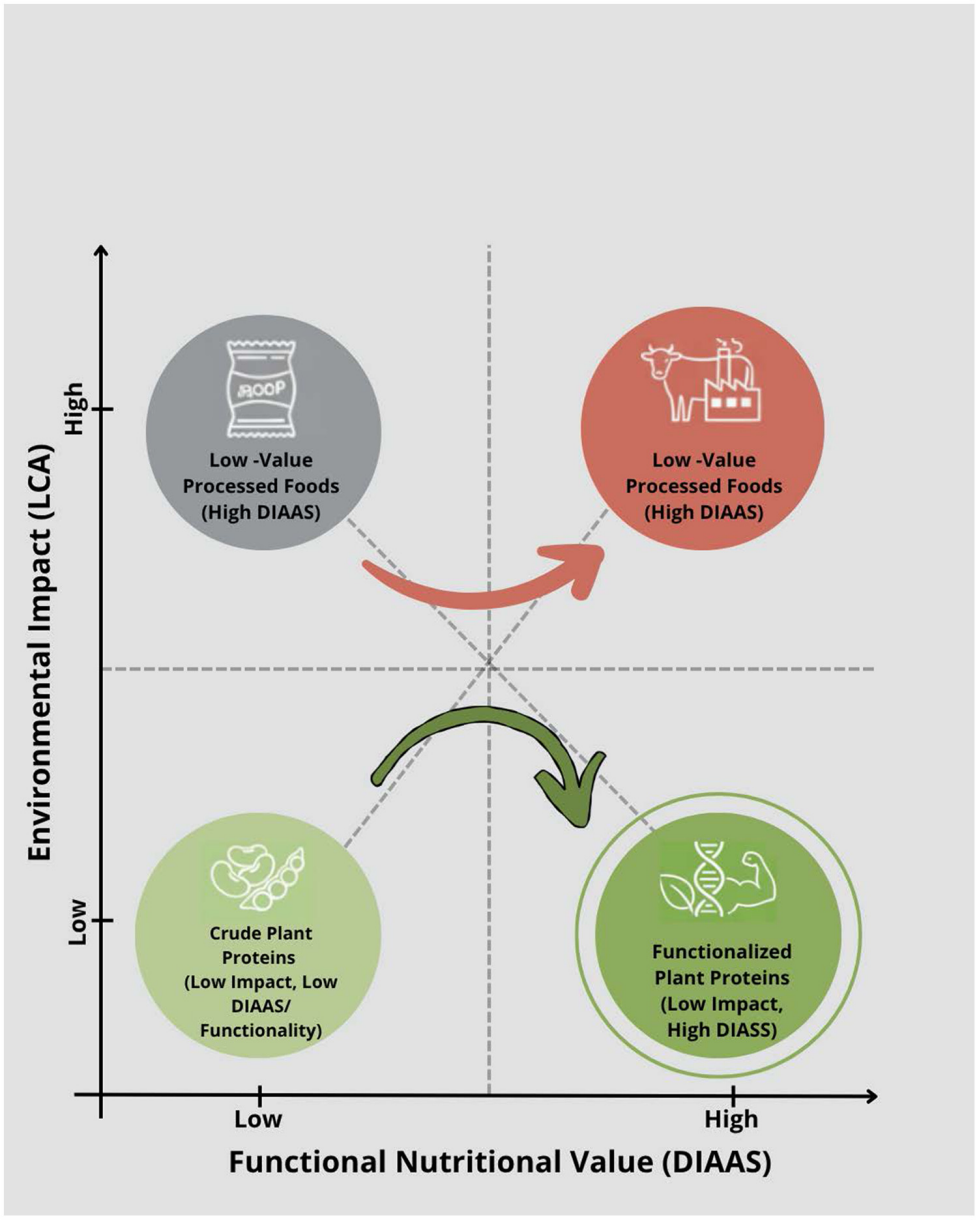
The intersecting mandates: balancing sustainability and nutritional quality.

Figure 1 illustrates the dual market pressure: the goal is to align low environmental impact, assessed via Lyfe Cycle Assessment (LCA) with maximum nutritional benefit, validated via Digestible Indispensable Amino Acid Score (DIAAS).

While vegetable sources like legumes and cereals offer compelling advantages, including an appealing amino acid profile and significantly reduced ecological footprint compared to animal sources (8), their successful large-scale integration into complex, appealing food systems presents substantial technological hurdles. Overcoming these limitations is paramount to meeting the soaring consumer demand for palatable, effective, and sustainable alternative proteins (9). The evolution of the food system has therefore positioned protein-fortified foods, particularly those utilizing vegetable protein isolates (10), as a fundamental component of the modern diet. Comprehensive systematic reviews and meta-analyses consistently highlight the contribution of plant-derived proteins to the prevention of numerous chronic diseases and overall wellness (11, 12), affirming their position as a superior choice for promoting long-term health (13). However, the large-scale formulation of these new products is not without significant obstacles.

The successful commercialization relies heavily on achieving parity, or superiority, with conventional products in terms of consumer experience. Herein lie the technological challenges, primarily related to the often-negative impact of native plant proteins on crucial sensory properties and desired texture (9). These functional limitations represent a crucial barrier to widespread acceptance. Issues such as residual off flavors (often described as “beany” or “herbaceous”), a perceived chalkiness or grittiness, reduced solubility, and suboptimal gelling or emulsifying capacity are inherent to plant protein structures. The necessity of overcoming these sensory challenges is the decisive element for large-scale adoption. The long-term success of this transition is in fact linked not only to innovation, but also to the industry's ability to overcome the psychological and cultural barriers of the consumer, ensuring that the plant alternative does not require a sacrifice in quality or taste. Understanding the role of perceived personal relevance in motivating the consumer to reduce meat intake is fundamental to guiding the development of products that fully satisfy expectations without compromising eating pleasure (14). The competitive landscape also necessitates clearly addressing the current challenges that alternative proteins face, from the sensory to the economic side, to ensure they are not perceived as niche products, but as the future mainstream of global food systems, offering scalable, affordable, and widely accepted solutions that can meet the nutritional demands of a growing population while reducing environmental impact (15).

Plant proteins can sometimes be incomplete, meaning they may be low in one or more essential amino acids. However, this problem is easily overcome through a concept called protein complementation. To overcome it, it's possible to combine different plant protein sources. Different plant foods lack different amino acids. By eating a variety of them, you can get all essential amino acids over the course of the day. Grains (like rice, wheat) are low in lysine but higher in methionine; legumes (like beans, lentils) are high in lysine but low in methionine therefore when eaten together, they complement each other. Some examples can be represented by rice and beans, peanut butter and whole wheat bread; lentils and roti; hummus and pita. It is not necessary to combine foods in the same meal. Eating a variety of plant proteins across the day, the body can pool the amino acids and use them effectively. It is also worth considering that some plant foods as soy (tofu, tempeh), quinoa or buckwheat already contain all essential amino acids, therefore they represent another alternative to include complete plant proteins in the diet.

To successfully bridge the gap between nutritional promise and industrial reality, research is intensely focusing on targeted modification strategies aimed at radically improving the functionality and overall acceptability of vegetable proteins (16, 17). These innovative approaches, that include physical, chemical, enzymatic, and biotechnological interventions, are designed to precisely engineer the structure and performance of plant protein isolates (18). The native functionality of most plant proteins, particularly those from legumes, is limited by their molecular configurations, which are often insoluble and tightly folded. To make them versatile ingredients, capable of creating stable emulsions, aerated foams, or fibrous matrices (as in meat analogs), it is essential to modify their structure. Physical modifications, such as high-moisture extrusion or high-pressure treatment, alter the protein conformation, often partially denaturing them to expose hydrophobic groups and improve interaction with water and fat. This process is crucial and has been the subject of recent, in-depth reviews detailing the precise mechanism by which physical treatments transform the functional properties of plant proteins (19). Concurrently, chemical and enzymatic approaches aim to break down or recombine protein bonds. For example, using enzymes like transglutaminase improves the gelling capacity and strength of the protein network, which is essential for the texture of products like plant-based cheeses or meats. Biotechnology and fermentation are also instrumental, offering methods for de-flavoring and improving solubility through the production of smaller, more water-soluble peptides. Furthermore, the aspect of digestibility and bioavailability remains paramount. While plant proteins possess an interesting amino acid profile, factors such as anti-nutritional compounds and the tightly folded structure of native proteins can impede enzymatic access, thereby reducing the effective absorption of amino acids. Targeted processing techniques, encompassing recent advances in protein modification methods (20), are instrumental in mitigating these factors, ensuring that the fortified products deliver their promised nutritional value (21). Therefore, the pursuit of new processing technologies, from biotechnology to food engineering, is the engine of innovation, as it aims to unlock the full nutritional and functional potential of plant proteins.

As regards plant-based products, basic foods as lentils, beans, chickpeas, tofu, soy or grains are almost always cheaper than meat, fish, or dairy per kg or per serving. For example, in Europe, legumes can cost € 1–3/kg, making them among the cheapest protein sources. Therefore, a simple plant-based diet can be very budget-friendly. On the other side, processed plant-based products are often more expensive. Plant-based burgers or sausages, meat analogs and vegan cheese or milk alternatives are often more expensive than animal products, especially in Europe. For example, plant-based meat alternatives can cost 20%−30% more than regular meat because big meat industries benefit from decades of optimization and subsidies, which plant-based producers often don't have. More than one reason can be cited: more processing, smaller production scale and use of specialized ingredients. However, the trend is changing because prices of plant-based products are going down over time because processed plant-based alternatives behave more like tech-driven products than raw agriculture.

## Cereal-based products fortified with vegetable proteins

The evolution of consumer demand has established cereal-based products, including high-volume staples like pasta, bread, and various breakfast and snack items, as a primary and highly accessible vehicle for protein enrichment, particularly through the use of vegetable proteins. Historically, cereals, while rich in carbohydrates, are nutritionally limited by the lower content and quality of their native protein, often lacking essential amino acids like lysine. The strategic incorporation of protein concentrates or flours derived from legumes, known for their complementary amino acid profile, serves a critical nutritional function, elevating the overall Protein Digestibility Corrected Amino Acid Score (PDCAAS) of the final product. Legumes such as lentils, chickpeas, and, most prominently, peas, are frequently employed due to their neutral flavor profile, cost-effectiveness, and added benefit of increasing dietary fiber, a crucial factor in maintaining gut health. Research has focused on pasta, where the addition of chickpea or lentil flours improves the nutritional profile and fiber content, while simultaneously altering the physicochemical and sensory properties of the final product (22–25). Studies have explored how fortification influences the texture, color, and cooking properties in diverse pasta varieties, including spaghetti and instant noodles (26–28). Beyond pasta, the baking and snacks sector has also seen a significant wave of innovation. The integration of vegetable proteins, such as those from pea, has enabled the development of high-protein, low-carbohydrate products, which inherently present unique challenges related to texture and consumer acceptance (29, 30). Research has explored the functional properties of different protein concentrations and their effects on the structure of bread and muffins, with particular attention to gluten-free formulations (31–33). Extruded snacks have similarly benefited from this trend, with studies analyzing the impact of barley and lentil flour blends on their physical and microstructural properties (34).

The successful implementation of fortification strategies fundamentally depends on the functional properties of the protein ingredient. Legume proteins, primarily storage globulins (such as vicilins and legumins), must exhibit high water holding capacity, gelling, and emulsification capabilities to integrate successfully into the cereal matrix. Unlike wheat proteins (gliadins and glutenins), which form the coherent viscoelastic network known as gluten, vegetable globulins possess molecular structures that often hinder effective functional integration. Therefore, a detailed understanding of the characterization and specific functional properties of legume protein isolates, often influenced by their processing methods (e.g., wet isolation vs. dry milling), is the key to optimizing their use (35). The degree of purity and denaturation resulting from extraction profoundly alters the solubility and the accessibility of hydrophobic groups essential for interaction with other dough components. The evaluation of functional properties, such as swelling capacity and gel consistency, of legume flours is crucial for determining their suitability; for example, flours with high water absorption capacity can aggressively compete with native starch and proteins for moisture, negatively influencing the final texture of baked goods by making them drier or less voluminous (36). The primary goal for formulators is thus to find the optimal saturation point that effectively balances nutritional gains without severely degrading the essential rheological properties necessary for processing and maintaining sensory acceptance.

**Figure 2:**
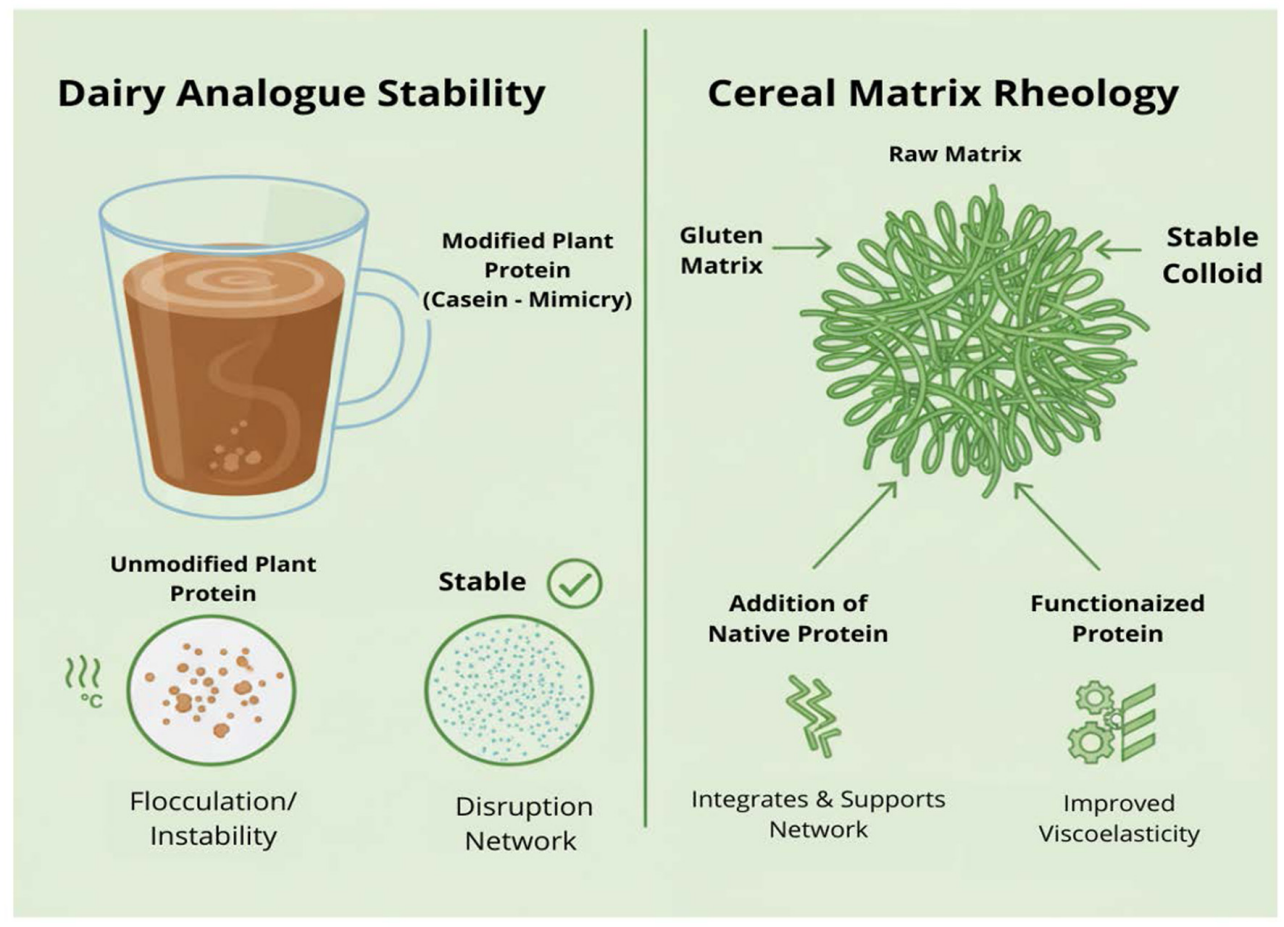
Functional challenges of plant proteins in complex matrices: stability and rheology.

The impact of adding vegetable proteins on the rheology of traditional pasta and baked goods remains the principal technical barrier. In both systems, the incorporation of legume proteins (typically substituting 10%−20% of the wheat flour) leads to a general weakening of the gluten network. In durum wheat pasta, structural stability is paramount, as gluten provides the necessary extensibility and cohesion to minimize the unacceptable loss of solids during cooking (cooking loss). Vegetable globulins act as largely inert fillers, physically impeding the formation of essential disulfide bonds within the gluten proteins. This disruption commonly results in both increased cooking loss and suboptimal, pasty, or chalkier texture after cooking, due to protein matrix dilution and poor water retention. This challenge is so significant that advanced research focuses on modifying the proteins before their inclusion. For instance, the use of enzymes like chymosin on pea protein isolates (*Pisum sativum*, L.) has been proven to alter the native protein structure, successfully enhancing its binding properties and significantly mitigating the negative impact on texture and cooking loss in food models (37). Such targeted modifications are critical because the native functional characteristics of legumes (such as minimal solubility near their isoelectric point, pH 4–5) are often inherently counter-productive to the rheological requirements of pasta making, where cohesion and extensibility are vital. Figure 2 schematically illustrates these functional failures, depicting how unmodified protein molecules can aggregate under processing stress (flocculation) or physically interfere with the cohesive structural network of the dough.

In baking, the gluten rheology is even more sensitive. Vegetable proteins exert a detrimental effect in two primary areas: reduced loaf volume, where unbound protein particles interfere with the network's ability to retain carbon dioxide produced during leavening, thereby limiting oven spring; and altered crumb structure, where the internal matrix becomes denser and less uniform due to the physical disruption of the gas cells. To counteract these rheological failures, the food industry relies on techniques such as ultrafine milling of legume flours to reduce particle size, thus minimizing physical interference with the gluten matrix. Additionally, the use of functional additives like enzymes (e.g., transglutaminase) and hydrocolloids (e.g., hydroxypropyl methylcellulose) is standard practice to artificially reinforce the dough matrix. These additives stabilize air bubbles and enhance viscosity, effectively compensating for the lost structural function of the gluten network.

The burgeoning market for gluten-free products presents a unique context where vegetable proteins are not merely fortifiers but crucial structural substitutes. Since these products rely on starches and alternative hydrocolloids, the protein must actively contribute to the structure, providing essential gelling, emulsifying, and water-binding functions to compensate for the absence of gluten. Achieving the desired porosity in gluten-free bread or the required firmness in pasta demands a precise balance of pea, rice, or potato protein isolates to mimic the viscoelastic properties normally provided by the gluten (9, 10).

Beyond textural quality, a critical area of focus is ensuring the nutritional efficacy of the fortified proteins, particularly their bioavailability. Despite their high crude protein content, pulse proteins often suffer from reduced digestibility due to their complex globular structure and presence of Anti-Nutritional Factors (ANFs) like phytates and protease inhibitors. Phytic acid, for instance, chelates essential minerals, while protease inhibitors (such as trypsin and chymotrypsin inhibitors) block the body's digestive enzymes, further limiting protein breakdown. From a nutritional perspective, effective fortification requires that processing steps (such as specific hydrothermal treatment, controlled fermentation, or extrusion itself) must be optimized to inactivate these ANFs and denature the protein, thereby rendering it more accessible to proteolytic enzymes (21). In addition to metabolic efficiency, the physiological impact on appetite regulation represents a primary benefit of this synergetic combination. Increasing the protein density of everyday foods is a key strategy for enhancing satiety and managing food intake. Protein is recognized for its superior effect on promoting fullness compared to carbohydrates or fat, regulating appetite via hormonal feedback mechanisms involving the release of gastrointestinal peptides like Cholecystokinin (2). The relationship between these two sources is bidirectional: while legumes provide the specific bioactive peptides that trigger satiety signals, the cereal matrix acts as a familiar and accessible vehicle for their delivery. Furthermore, the protein leverage hypothesis suggest that organisms seek to maintain a target protein intake; if diet is diluted in protein, they increase their total energy intake from fat and carbohydrates to compensate (3). However, to translate these nutritional advantages into successful products, the underlying structural mechanism must be addressed. The fundamental conflict remains the interaction between legume globulins and the cereal gluten network. From a functional and textural standpoint, the way these proteins dock within the matrix determine the final quality. Emerging non-thermal physical modification techniques are gaining prominence for fine-tuning protein functionality without resorting to chemicals. Methods such as ultrasound and high-pressure processing are being explored to mechanistically unfold the protein globulins, enhancing their solubility, emulsifying properties, and thermal stability before they are introduced into the dough matrix, potentially solving many of the afore-mentioned rheological issues (16, 19). In summary, while cereal-based products offer an ideal pathway for mass protein enrichment, the technology must continuously overcome the fundamental conflict between the pursuit of high nutritional value and the imperative of maintaining the expected rheological and sensory properties. The balance between nutritional augmentation and functional integrity requires matrix-specific engineering strategies. Table 1 provides a concise overview of representative approaches across diverse cereal products, detailing the primary objectives, the specific protein source utilized, and the technological or sensory challenges that currently remain unresolved.

**Table 1:** Engineering strategies and functional challenges of plant protein fortification in cereal matrices.

Cereal matrix	Plant protein source	Engineering strategy/treatment	Primary functional/technological objective	Unresolved technological or sensory challenge	References
Pasta (durum wheat semolina)	Chickpea isolates/flours	Dry mixing techniques (simple substitution)	Increase the overall protein content (up to 20%) while minimally disrupting the native gluten network integrity.	Significant alteration of product color (browning) and increased cooking loss due to structural weakness.	(46)
Pasta/noodles	Pea protein	High-density pressurization (HDP)	Improve the structural stability and thermal properties of the noodles to reduce solids dispersion during cooking.	Maintaining the native elastic texture of the cooked product and mitigating the sensory perception of *chalkiness*.	(20)
Wheat bread	Soy protein isolate (SPI)	Enzymatic modification (protein-glutaminase)	Enhance the strength and stability of the protein network by promoting cross-linking and improving gas retention.	Preventing the specific loaf volume collapse and avoiding an overly dense or compact crumb structure.	(63)
Baked goods (biscuits, crackers)	Lupin flour/concentrates	Partial flour substitution (optimization)	Improve the nutritional profile (protein and fiber) and modulate the water-holding capacity for desired hardness/texture.	Development of pronounced bitter or *beany* off-flavors at substitution levels exceeding 15%.	(64)
Extruded products (snacks, meat analogs)	Pea and wheat proteins	High moisture extrusion (HME)	Induce protein reorganization and aggregation to achieve a defined fibrous, anisotropic structure (*meat-like texture*).	Precise control over the thermal and shear history to prevent protein denaturation and loss of solubility/functionality.	(46)
Gluten-free products (rice, maize)	Quinoa (pseudocereal)	Nutritional characterization/processing	Optimizing the utilization of pseudo-cereals to maximize functional components and effectively manage anti-nutritional factors (ANFs)	Balancing the efficient removal of anti-nutritional compounds (e.g., saponins) with the retention of beneficial functional/bioactive components.	(65)
Functional flours	Oilseeds (hemp, flax)	Ultra-fine milling	Increase specific surface area for superior emulsification capacity and foam stabilization in batter/dough systems.	Mitigating lipid oxidation (*rancidity*) and effectively masking the associated strong, herbaceous flavors.	(49)

## Plant-based products fortified with vegetable proteins

The surging demand for plant-based products has intensely driven the food industry to focus on protein fortification and creation of entirely animal-free analogs, aiming not only to enhance nutritional profiles but, critically, to improve sensory attributes, which are recognized as the primary factor dictating consumer acceptance (9). A key area of innovation is the large-scale development of meat analogs, where proteins sourced primarily from legumes, such as pea and fava bean, are utilized to replicate the complex texture, mouthfeel, and savory flavor of conventional meat (38–40). Advanced technologies like High Moisture Extrusion (HME) are essential for creating the required fibrous and anisotropic structure for these products, which are then tested in formulations such as hybrid or entirely plant-based burgers to evaluate their physicochemical properties and market acceptance (41–43). Furthermore, vegetable proteins are also employed to fortify other food matrices, such as smoothies and functional beverages, specifically to improve their nutritional and sensory characteristics (44). Consumer acceptance and sensory properties ultimately remain the principal factors driving innovation across this entire sector (45).

The process of replicating muscle architecture, mimicking the structural components of meat, represents the most demanding technical hurdle, requiring the precise transformation of spherical plant globulins into highly aligned fibers. The success of HME hinges on its ability to subject a high-moisture, protein-rich dough (typically 40%−70% water) to a combined treatment of high temperature (~120 °C−180 °C), pressure, and intense mechanical shear forces within a heating barrel, followed by rapid cooling in an elongated die (46). This thermo-mechanical action induces protein denaturation (unfolding of the native structure) and immediate re-polymerization into a layered, aligned structure, which is fundamental to imparting the fibrous index and chewiness expected by the consumer. Extensive research has meticulously mapped the relationship between extrusion parameters and resulting texture. The moisture content is critical: lower moisture levels lead to higher mechanical energy input and better protein alignment, but risk burning or excessive denaturing. The temperature gradient in the long cooling die dictates the speed of protein re-assembly; a slower cooling rate can result in a less defined fibrous structure. The shear rate and the configuration of the screw elements determine the alignment of the protein molecules. A detailed review on high moisture extrusion highlights how the interaction between different plant protein types (e.g., soy, pea, and wheat gluten) and the internal forces generated during the process is the primary mechanism governing the formation of this layered, meat-like structure (46). Optimized parameters allow manufacturers to tailor the texture to replicate specific animal products, differentiating between the fine, easily separable fibers of poultry and the coarser and tougher structures required for beef analogs. The technical demands of replicating animal product functionality, whether the fiber of meat, the stability of dairy, or the flake of fish, necessitate a broad range of highly specific engineering and formulation strategies. Table 2 summarizes the key objectives, the critical treatments applied, and the persisting technological and sensory hurdles across these different plant-based food matrices.

**Table 2:** High moisture extrusion: mechanism and challenges for plant-based analogs.

Food matrix	Plant protein source	Engineering strategy/treatment	Primary functional/technological objective	Unresolved technological/sensory challenge	References
Meat analogs	Legumes (e.g., pea, fava bean)	High Moisture Extrusion (HME): thermo-mechanical action inducing denaturation and re-polymerization.	Replicate the complex fibrous and anisotropic structure; impart chewiness.	Replicate the succulence and mouthfeel of animal fat.	(46)
Meat analogs	Legumes (e.g., pea, fava bean)	High moisture extrusion (HME)	Replicate the complex fibrous and anisotropic structure; impart chewiness.	Optimize the balance between fibrosity (alignment) and cohesion of the final product.	(41)
Meat analogs	Legumes (e.g., pea, fava bean)	Formulation with functional additives (e.g., hydrocolloids) and fat mimetics.	Ensure effective fat emulsification and retention to simulate juiciness.	Overcome functional limitations of single-source proteins; encapsulate fat alternatives to replicate melting point and flavor.	(47)
Dairy analogs	Pea, rice, others	Physical modifications (e.g., ultrasound, high-pressure homogenization).	Improve emulsifying properties by altering protein surface structure.	Improve emulsifying properties, which are often inferior to dairy proteins.	(16)
Dairy analogs	Pea, rice, others	Enzymatic/physical modifications	Improve solubility and thermal/pH stability (prevent flocculation).	Replicate the thermal and pH stability of caseins. Protect against anti-nutritional factors (ANFs) and increase bioavailability.	(20)
Fish analogs	Artichoke, pumpkin, zucchini, broccoli, carrots, flax and chia seeds and algae residue	Shear cell technology.	Replicate a more lamellar or flaky structure.	Achieve a structure that disintegrates flakily upon chewing, rather than shredding.	(49)
Fish analogs	Legumes (soy, peas lentils, chickpeas),	Gelation and protein aggregation techniques.	Replicate a more lamellar or flaky structure.	Mastering protein aggregation techniques for the flaky texture.	(48)
Fish analogs	Algae	Incorporation of microalgae extracts (e.g., *Schizochytrium* species).	Deliver omega-3s (EPA and DHA) and contribute to the “marine” flavor.	Stability and non-oxidative encapsulation of sensitive marine-derived oils.	(50)
Generic fortified foods	Legumes	General formulation.	Enhance nutritional profile and sensory characteristics.	Consumer acceptance and sensory properties remain the principal driving factors for innovation.	(45)

Beyond HME, the success of meat analogs relies heavily on sophisticated formulation strategies that move beyond the basic protein fiber. This involves the critical addition of functional additives and fat mimetics to achieve succulence, mouthfeel, and desirable flavor release, which are often lacking in simple extruded bases. Plant proteins inherently bind water tightly but struggle to retain emulsified fat, leading to a dry, spongy texture. Current research and development efforts are intensely focused on optimizing the blend of various protein sources, non-protein functional components, and technological processing methods. This comprehensive approach is necessary to overcome the functional limitations of single-source proteins and ensure effective fat emulsification and retention, a key factor in simulating the juicy quality of animal fat (47). For example, the incorporation of specific hydrocolloids, such as methylcellulose, is crucial for binding water and fat within the protein matrix, preventing excessive moisture loss during cooking and providing the desired structural integrity and bite. The textural goal is to mimic the anisotropy of meat muscle bundles while achieving a satisfactory fracture ability and chewiness that closely matches the animal counterpart. The challenge of creating encapsulated fat alternatives that replicate the melting point and flavor contribution of animal fat is a major frontier in this sector.

However, the category of fortified plant-based products is not limited to fibrous meat substitutes. Dairy analogs (milk, yogurt, cheese) require an equally complex level of protein functionality. Here, the protein ingredient must act as an excellent emulsifier to maintain colloidal stability (essential in plant milk, particularly when mixed with coffee or subjected to heat) or as a gelling and coagulating agent (in yogurt and cheese analogs). The immense challenge is to replicate the thermal and pH stability of milk caseins across an acidic range without flocculation, a problem that has driven researchers to modify plant proteins (e.g., pea or rice) to improve their solubility and thermal stability. Research is highly concentrated on enzymatic and physical modification techniques to alter the native protein conformation, thereby enhancing its interaction with water and fat. This necessity for structural modification is universal across all applications and extends to protecting against anti-nutritional factors (ANFs) and increasing bioavailability in dairy substitutes, ensuring that technological innovation also enhances the actual nutritional value (20). Physical modifications, such as ultrasound and high-pressure homogenization, are vital for refining particle size and altering the protein surface structure to improve emulsifying properties, which are often inferior to those of dairy proteins (16, 20). The fish analogs sector also requires a different approach, demanding lamellar or flaky structures rather than fibers; here, success depends on mastering specific protein aggregation techniques, such as shear-cell technology or gelation, to archive a texture that disintegrates flakily upon chewing (48, 49).

In summary, the classification of these products must reflect this technological heterogeneity: while extrusion is the key mechanism for structured meat substitutes, the fortification of non-structured matrices relies on formulation strategies and enzymatic or physical modification aimed at ensuring sensory acceptance and stability without compromising the product's original rheology (50).

## Dairy products fortified with vegetable proteins

The application of vegetable proteins in the dairy sector represents a crucial direction of innovation, aimed both at the fortification of traditional products (to elevate their nutritional value) and the extensive development of entirely plant-based alternatives, commonly known as dairy analogs (51). The technological challenges involved are inherently complex, as the addition of vegetable proteins can profoundly alter the essential sensory and functional properties of dairy systems.

A significant area of research concerns yogurt, where protein enrichment can impact stability, texture, and flavor. Recent studies have explored the use of vegetable proteins to improve the structural stability of products like oat yogurt and to evaluate their impact on the physical, chemical, and microbiological characteristics of traditional dairy yogurt (52, 53). In developing plant-based yogurt alternatives, the technological focus shifts entirely to coagulation and gelation. Traditional yogurt relies on the acid coagulation of casein micelles, which form a cohesive gel network; conversely, plant proteins do not coagulate in the same manner. To successfully replicate the structure and thixotropic texture of yogurt, formulators must skilfully utilize hydrocolloids (such as starch or pectin) in combination with the protein isolate to form a stable gel during lactic acid fermentation. Studies examining the effect of plant protein enrichment show that specific concentrations significantly influence key quality parameters like syneresis (whey separation), viscosity, and overall sensory properties. Inadequate protein dispersion or unfavorable interaction with stabilizers can result in a grainy or chalky texture, severely compromising consumer acceptance (53).

Similarly, in the cheese sector, the integration of vegetable proteins is crucial for enhancing both nutritional and functional profiles. Research focuses on developing fortified dairy cheese, such as low-fat mozzarella enriched with soy or pea hydrolysates, studying their structural and functional properties during aging (46). For entirely plant-based cheese alternatives, the challenges concentrate on creating functional matrices capable of replicating the key characteristics of traditional cheese, such as meltability, shred-ability, and stretching ability. Recent studies analyse the physicochemical properties of plant-based alternatives fortified with calcium and the engineering of pea protein-based matrices for fermented cheese (54).

For plant-based beverages (milks), the fundamental problem is emulsion stability. Vegetable proteins (e.g., oat, soy, pea) must act as efficient emulsifiers to prevent phase separation and flocculation during storage. The challenge is particularly acute when products are exposed to extreme acidic or thermal conditions (such as being added to hot coffee), where the isoelectric point (pI) of vegetable globulins can lead to rapid precipitation. The transformation of plant globulins into dairy-functional components involves overcoming a trifecta of hurdles: mimicking caseins' coagulation, stabilizing complex emulsions, and achieving neutral flavor profiles. Table 3 distills the core functional challenges and the engineering strategies employed across the primary dairy analog and fortification segments, underscoring the gap between current technology and the gold standard of bovine dairy.

**Table 3:** Functional hurdles in dairy analogs: stability, emulsion, and gelation.

Food matrix	Plant protein source	Engineering strategy/treatment	Primary functional/technological objective	Unresolved technological/sensory challenge	References
Yogurt (traditional)	Hemp, pumpkin seed flour, pea protein isolate, wheat gluten, soy protein isolate	Protein enrichment; specific concentration adjustments.	Improve structural stability (e.g., in oat yogurt); evaluate impact on physical, chemical, and microbiological characteristics.	Control of syneresis (whey separation); achieving optimal viscosity; avoiding grainy/chalky texture.	(52)
Yogurt (plant-based)	Chickpea, pea	Formulation with hydrocolloids (starch, pectin) and protein isolate; lactic acid fermentation.	Replicate the coagulation and gelation network; simulate the thixotropic texture of dairy yogurt.	Inadequate protein dispersion or unfavorable interaction with stabilizers, leading to poor texture; masking off-flavors.	(53)
Cheese (dairy fortified)	Soy or pea hydrolysates	Fortification; structural and functional property studies during aging.	Enhance nutritional profile (e.g., low-fat mozzarella); maintain structural and functional properties during aging.	Maintaining desirable structural and functional properties (texture, moisture retention) during processing and storage.	(46)
Cheese (plant-based)	Pea protein, others	Matrix engineering; fortification with calcium.	Create functional matrices that replicate key characteristics: meltability, shred-ability, and stretching ability.	Replicating the exact functionality and texture of traditional cheese during heating and handling.	(54)
Plant-based beverages (milks)	Oat, soy, pea, others	Optimizing pH; high-pressure homogenization; inclusion of specialized hydrocolloids.	Ensure emulsion stability; Prevent phase separation and flocculation during storage.	Preventing rapid precipitation when exposed to acidic or thermal conditions (e.g., hot coffee); achieving long-term stability without separation.	(66)
Dairy analog sector (overall)	Peanut, soy, oat	Targeted enzymatic modification (for beverages); nutritional fortification.	Achieve solubility and stability equivalent to bovine milk across wide environmental conditions; enhance bioavailability of key nutrients.	Complete masking of earthy or beany off-flavors; effective fortification with bioavailable calcium and vitamin D.	(51)

Finally, for both beverages and yogurts, the necessity for effective nutritional fortification (with bioavailable calcium and vitamin D) and the complete masking of earthy or beany off flavors remain critical barriers to mass market adoption.

## Meat product fortified with vegetable

The incorporation of vegetable proteins into traditional meat products is a rapidly evolving area of research, driven by the multifaceted need to improve nutritional profiles, enhance sustainability, and optimize product functionality. This approach extends beyond the simple reduction of meat content, strategically utilizing vegetable proteins as true “extenders” to optimize the overall composition and functional properties of the final hybrid product (55, 56). Their use is critical for improving key technological attributes such as water retention capacity, texture, and slicing properties, as evidenced by studies focusing on non-meat alternatives and hybrid formulations (9, 57). This strategy of fortification is particularly attractive because it allows manufacturers to leverage the familiar sensory appeal of meat, while addressing environmental and cost concerns.

Research has focused significantly on specific high-volume products such as beef burgers. Studies have demonstrated that the strategic addition of proteins derived from legumes, such as pea or soy, and even microalgae can significantly enhance both the technological and qualitative characteristics of the final patty (58). In particular, fortified burger formulations have been developed for specific consumer groups, such as the elderly, with the primary objective of increasing their protein intake in an appealing, familiar format, thereby mitigating age-related sarcopenia (59, 60). This principle of utilizing plant protein for nutritional and functional enhancement has also been successfully extended to other processed products, such as chicken nuggets, where the inclusion of pea and rice protein isolates has been shown to improve texture, binding capacity, and overall sensory properties (61).

The functional role of vegetable proteins in these hybrid matrices is complex and highly dependent on their native properties and processing treatment. Unlike meat analogs, which demand maximum fibrosity (as detailed in previous sections), here the focus is on maximizing emulsification and binding capacity. Vegetable proteins, especially highly soluble isolates, act to stabilize the fat and water that are naturally released from the comminuted meat structure during cooking and processing. By efficiently binding water, these proteins reduce cooking loss and prevent the excessive shrinkage often associated with lean meat products, thereby ensuring a juicier, and more tender final product. Conversely, if the plant protein is poorly dispersed or lacks adequate water-binding capacity, it can lead to a chalky or dry mouthfeel, which is detrimental to consumer acceptance.

To effectively integrate the plant protein, the industry relies on careful preparation and selection of the protein type. For instance, hydrolysed soy or pea proteins are often preferred for their enhanced solubility and reduced tendency to precipitate, which improves integration into the meat matrix. The success of this hybridization strategy hinges on precisely engineered trade-offs, where functional requirements shift from fibrosity (as seen in analogs) to superior binding and emulsification. Table 4 provides a focused summary of these objectives, illustrating the dual pursuit of optimized functionality and enhanced nutritional delivery across different hybrid meat products.

**Table 4:** Hybridization strategy: vegetable proteins as binders and extenders in meat.

Food matrix	Plant protein source	Engineering strategy/treatment	Primary functional/technological objective	Unresolved technological/sensory challenge	References
Hybrid meat products (general)	Legumes (soy, beans and others)	Strategic inclusion/utilization as “extenders.”	Improve sustainability and optimize product functionality (texture, slicing properties).	Achieving an optimal balance where nutritional/economic gains do not compromise the familiar sensory experience (color, bite, juiciness).	(55)
Hybrid meat products (general)	Legumes, oilseeds, cottonseed and others	Incorporation into the comminuted meat structure.	Improve key technological attributes like water retention capacity and texture.	Preventing a chalky or dry mouthfeel due to poor protein dispersion or inadequate water-binding capacity.	(57)
Beef burgers	Legumes (pea, soy), microalgae	Strategic addition and formulation.	Enhance technological and qualitative characteristics (e.g., juiciness, reduced cooking loss).	Masking/removing inherent off-flavors (e.g., beany notes) carried by some plant protein sources.	(58)
Beef burgers (targeted)	Legumes (e.g., pea, soy)	Fortification for specific consumer groups (e.g., elderly).	Increase protein intake in an appealing format to mitigate age-related sarcopenia.	Maintaining the appealing, familiar format and taste associated with conventional burgers.	(60)
Chicken nuggets	Pea and rice protein isolates	Inclusion during processing.	Improve texture, binding capacity, and overall sensory properties.	Ensuring the protein is uniformly distributed (using equipment like high-shear choppers) without negatively affecting color or flavor.	(61)
Hybrid meat products (overall)	Hydrolysed soy/pea proteins	Preference for hydrolysates; use of advanced processing equipment (vacuum mixers).	Maximize emulsification and binding capacity to stabilize fat and water; reduce cooking loss and prevent excessive shrinkage.	The functional role of the protein must be carefully selected to maximize emulsification and not compromise texture.	(56)

Ultimately, the successful commercialization of these fortified meat products hinges on achieving an optimal balance where the nutritional gains from the plant protein and the economic benefits of using extenders do not compromise the sensory experience, particularly the color, bite, and juiciness, that consumers associate with high-quality conventional meat. This hybridization approach represents a sustainable and pragmatic pathway for improving the health footprint of a major segment of the global diet.

## Fish product fortified with vegetable proteins

The seafood industry is increasingly pivoting toward more sustainable and nutritionally advanced solutions, integrating vegetable proteins into traditional fish products. This trend is primarily driven by the imperative to reduce the significant environmental impact associated with conventional fishing and aquaculture practices, and to meet the growing consumer demand for healthier, more ethically sourced foods (9). Vegetable proteins are being used not merely for nutritional enrichment but also to crucially enhance the functional properties of processed seafood items. This dual role, nutritional supplement and functional optimizer, is vital for maintaining product quality while achieving cost and sustainability goals.

**Figure 3:**
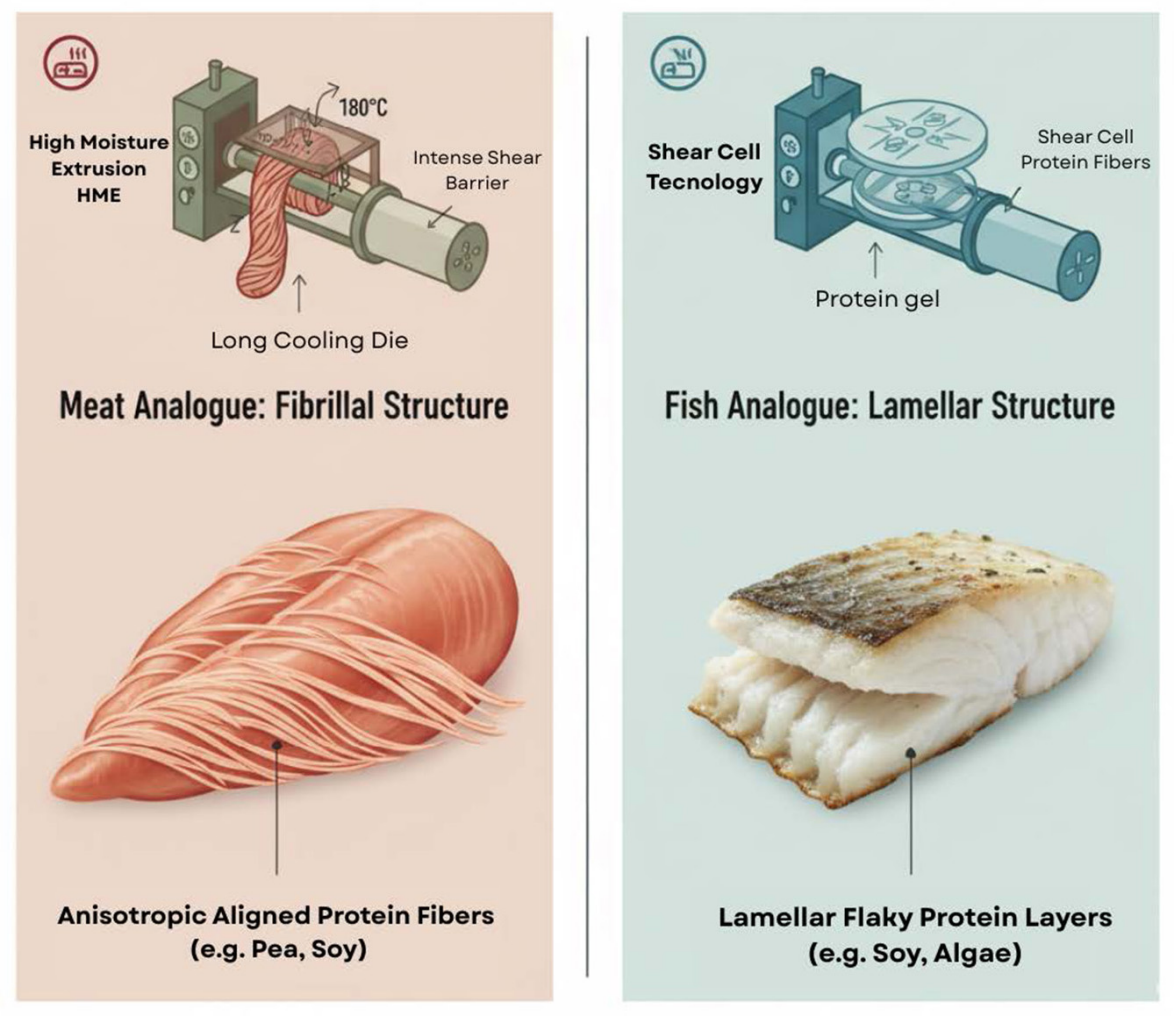
Structural engineering for analogs: fibrillar vs. lamellar textures.

A key area of innovation and research is the development of surimi gels and related comminuted seafood products. Surimi, a highly processed fish paste used in products like imitation crab meat, relies on the intrinsic gelling ability of muscle protein (myosin) to form a desirable elastic texture, known as set or gel strength. The addition of vegetable proteins, particularly those from chickpeas, is being extensively studied to enhance these structural properties. These plant proteins improve gelling capacity, reinforce texture, and critically act as a cryoprotectant in frozen seafood products (20, 62). During freezing and thawing cycles, vegetable proteins can effectively bind water, minimizing the formation of large ice crystals that damage muscle fibers and cause unacceptable texture loss (Figure 3). These innovative ingredients allow manufacturers to produce final products with optimal sensory and nutritional attributes, even with a reduced content of high-cost fish muscle (20).

In parallel, the sector is rapidly advancing the development of entirely plant-based fish analogs. The objective here is far more ambitious: to faithfully replicate the complex texture, subtle flavor, and high nutritional value of both white fish and seafood (e.g., shrimp, tuna) using exclusively vegetable proteins. This is a rapidly expanding field that opens new market frontiers, differentiating itself from meat analogs due to the specific textural challenges. The dual focus on functional optimization in existing seafood (e.g., cryoprotection) and novel structural engineering for analogs defines this sector. Table 5 outlines the specific functional objectives and the cutting-edge strategies utilized to achieve structural, nutritional, and sensory parity in both fortified and fully plant-based fish products.

**Table 5:** Seafood innovation: from surimi cryoprotection to lamellar texture engineering.

Food matrix	Plant protein source	Engineering strategy/treatment	Primary functional/technological objective	Unresolved technological/sensory challenge	References
Processed seafood (e.g., surimi gels, comminute products)	Chickpeas, other vegetable proteins	Incorporation into muscle protein matrix.	Enhance gelling capacity and reinforce texture; act as a cryoprotectant to minimize ice crystal damage during freezing/thawing.	Achieving optimal sensory and nutritional attributes while reducing high-cost fish muscle content.	(20)
Processed seafood (e.g., surimi gels)	Vegetable proteins	Strategic addition and formulation.	Improve gelling capacity and reinforce texture (gel strength).	Ensuring vegetable proteins effectively bind water to prevent texture loss during freezing/thawing cycles.	(62)
Fish analogs (plant-based)	Various vegetable proteins	Specialized protein aggregation techniques: gelation and shear cell technology.	Replicate the lamellar, flaky, or soft texture of white fish muscle; achieve structure that disintegrates flakily upon chewing.	Mastering aggregation techniques to produce the flaky texture (distinct from the cohesive fiber needed for meat).	(48)
Fish analogs (plant-based)	Various vegetable proteins	Specialized protein aggregation techniques.	Replicate the lamellar, flaky, or soft texture; achieve structure that disintegrates flakily upon chewing.	Replicating the complex texture and subtle flavor of both white fish and seafood.	(49)
Fish analogs (plant-based)	Vegetable proteins and microalgae extracts (e.g., oil)	Nutritional enrichment and flavor engineering.	Achieve nutritional parity (high-quality protein, essential omega-3s); deliver the characteristic “marine” or “briny” flavor.	Ensuring the stability and non-oxidative encapsulation of plant-derived omega-3 fatty acids.	(9)

The final challenge is ensuring nutritional and sensory parity. Fish is prized for its high-quality protein and essential omega-3 fatty acids (Eicosapentaenoic acid, EPA) and (Docosahexaenoic acid, DHA). Therefore, successful analogs must be enriched with plant-derived omega-3 sources (e.g., microalgae oil) to match the nutritional profile. Furthermore, the characteristic “marine” or “briny” flavor must be achieved without relying on seafood components. Research is focused on utilizing novel fermentation techniques and microalgae extracts to deliver these complex flavor notes. The ability of vegetable proteins to serve as both functional enhancers in traditional surimi and the foundational material for next-generation, high-fidelity seafood analogs underscores their versatility and their essential role in the sustainable evolution of the global food supply (48, 49).

## Ecological and market integration

The ecological and market integration of plant-based products has become an increasingly important development in global food systems. From an environmental perspective, plant-based foods are often promoted as a more sustainable option because they generally require fewer natural resources and generate lower greenhouse gas emissions compared to animal-based production. Livestock farming, for example, is strongly associated with deforestation, methane emissions, and high-water consumption, while overfishing continues to threaten marine ecosystems and biodiversity. By contrast, producing plant-based proteins tends to have a smaller ecological footprint, making these alternatives attractive in the context of climate change mitigation and environmental conservation. At the same time, the integration of plant-based products into the market reflects both opportunities and challenges. In recent years, technological innovation has enabled the development of sophisticated substitutes that mimic the taste and texture of meat, fish, and dairy products, increasing their appeal to a broader range of consumers. This has supported the rapid expansion of the plant-based sector, with new companies entering the market and established food corporations investing heavily in alternative proteins. However, despite this growth, plant-based products often remain more expensive than conventional animal-based foods due to higher production costs, smaller economies of scale, and ongoing research and development expenses. Consumer perception also plays a crucial role in market integration. While awareness of environmental and ethical issues is rising, purchasing decisions are still influenced by price, taste, cultural habits, and accessibility. In many regions, traditional diets are deeply rooted in animal-based foods, which can slow the adoption of alternatives. Furthermore, policy frameworks and subsidies continue to favor conventional agriculture in many countries, limiting the competitiveness of plant-based options. To sum up, the transition toward plant-based alternatives involves a complex interaction between ecological benefits and market dynamics. While these products offer significant potential to reduce environmental impacts and diversify food systems, their widespread adoption will depend on continued innovation, supportive policies, and shifts in consumer behavior.

## Conclusion and future outlook

A critical analysis of the current literature, as summarized in the tables of this review, highlights that despite significant progress, several unresolved technological and sensory challenges still define the primary research gaps in plant protein application:

- Texture and structural integrity in baked goods and pasta: The tables highlight how simple substitution with legume proteins often weakens the gluten network, leading to increased cooking loss in pasta or loss of loaf volume and dense crumb structures in bread. To address this gap, the use of targeted enzymatic modifications, such as treating pea protein isolates with chymosin or using cross-linking enzymes like transglutaminase, has shown promise in repairing network deficiencies. Physical interventions like ultra-fine milling to reduce particle size have also been hypothesized to minimize physical interference with the continuous starch-protein matrix.
- Juiciness and fat retention in meat analogs: While High Moisture Extrusion (HME) successfully aligns plant globulins into fibrous structures mimicking meat muscle, replicating the succulence and mouthfeel of animal fat remains a persisting technological hurdle listed in Table 2. A clear solution gathered from literature is the transition from simple water-binding formulas to advanced fat-mimetics and encapsulation strategies. Combining specific hydrocolloids (e.g., methylcellulose) with plant proteins can trap plant-derived oils, ensure their controlled release and melting profile during cooking, thus bridging the gap toward true sensory parity. A representative commercial example of this strategy is found in product by Beyond meat or impossible Foods, which leverage combinations of pea or soy proteins with coconut and canola oils, bound by methylcellulose, to mimic the sizzle and juiciness of beef burgers.
- Flaky texture in seafood alternatives: As evidenced in the challenges for fish analogs, generating a lamellar, flaky structure that disintegrates upon chewing, rather than a tough, shredded one, remains highly problematic. Here, the research gap points directly to the limitation of traditional extrusion. Hypothesized and emerging solutions involve moving away from high-shear methods toward lower-shear or gelation- driven techniques such as shear-cell technology, which allows for more delicate protein aggregation and layered sheets instead of tight, longitudinal fibers. On the market, brands like Good Catch address this specific texture gap by utilizing a proprietary blend of legumes to successfully replicate the flakiness of tuna and white fish.
- Colloidal and thermal stability in dairy analogs: The tables showcase challenges regarding off-flavors and poor solubility resulting in chalkiness or flocculation, particularly in acidic or hot environments (e.g., plant milks in coffee). To overcome these limitations, precision engineering must leverage non-thermal physical modifications like high-pressure homogenization and ultrasound. These methods can unfold native, tightly packed globulins, exposing hydrophilic sites that enhance emulsification and keep the proteins in suspension, effectively mimicking the stability of milk caseins. In the current commercial landscape, Oatly serves as a prime example of overcoming these issues: through controlled enzymatic action on oat starch and targeted use of acidity regulators, they prevent separation in hot coffee, offering a functional alternative that matches dairy performance.

The trajectory of food innovation will concentrate on resolving the fundamental functional conflict between nutritional value and sensory acceptance through advanced scientific intervention. To achieve this, a structured framework of challenges and solutions is necessary. The central pillar of this revolution is the functionalization of plant proteins. Research is rapidly moving away from using raw protein isolates toward creating high-performance ingredients with tailored capabilities. This involves intensive modification, leveraging enzymatic treatments, high-pressure homogenization, and advanced bioprocessing to engineer specific functionalities. For instance, the future of plant-based dairy demands proteins capable of Casein-Mimicry exhibiting guaranteed pH and thermal stability to prevent flocculation in complex systems like hot coffee. Simultaneously, in the cereals sector, modified isolates will act as active structural aids, overcoming the rheological limitations imposed by gluten dilution, thus ensuring fortified baked goods maintain volume and texture quality. Achieving sensory parity will necessitate a hyper-focus on matrix and fat engineering. In the analog space, the challenge transcends simple fibrillization. Meat analogs will evolve through sophisticated high moisture extrusion that identifies a typology of technologies and predictive rheological models to replicate specific cuts of muscle, rather than generic fiber. The crucial differentiation lies in the seafood sector, where shear cell technology and controlled gelation will be paramount for generating the unique, lamellar, flaky texture required by fish analogs. This pursuit of textural fidelity is inextricably linked to Next-Generation Fat Engineering, where plant-based oleo gels and structured emulsions will be designed with controlled melt points, finally solving the industry-wide problem of dryness and ensuring authentic succulence and flavor release upon consumption.

This technological evolution is intrinsically tied to nutritional accountability. The industry is shifting from measuring crude protein content to verifying the actual biological value. The DIAAS will become the standard for nutritional claims, necessitating optimized processing steps (such as specific hydrothermal treatments) that neutralize Anti-Nutritional Factors and maximize bioavailability. This rigor extends to new product categories; for instance, marine analogs will routinely incorporate encapsulated microalgae-derived Omega-3s to ensure full nutritional equivalence.

The broad adoption of these advanced products will be underpinned by strategic market integration and transparency. Critically, the continued success of the plant protein paradigm requires focused research on the following critical points:

- Nutritional Validation Rigor: Establishing standardized methodologies for the widespread use of DIAAS as the nutritional benchmark, coupled with optimized processing to routinely neutralize Anti-Nutritional Factors and maximize protein bioavailability.
- Matrix and Fat Engineering: Advancing sophisticated HME 2.0 and shear cell technologies for the precise replication of complex analog textures, alongside the development of Next-Generation Fat Engineering (oleo gels/structured emulsions) to control melt points and enhance succulence and flavor release.
- Casein-Mimicry and Functional Stability: Prioritizing the development of modified isolates capable of robust Casein-Mimicry, exhibiting guaranteed pH and thermal stability to prevent flocculation in complex plant-based dairy systems.
- Ecological and Market Integration: Underpinning product adoption with the standardized and transparent use of LCA data to unequivocally prove environmental superiority, supporting the strategic positioning of fortified and hybrid products as functional foods targeting specific demographics.

The transition toward a truly sustainable and healthy global diet hinges on the success of Precision Protein Engineering, necessitating a unified effort across molecular biology, food chemistry, and engineering to realize the full potential of plant-based proteins.

In conclusion, moving forward from these identified gaps requires a shift from empirical testing to precision molecular design. The future of plant-based foods will not be dictated by the quantity of protein added, but by the technological capability to restructure and functionalize these proteins to fit perfectly within specific food matrices.
